# Simultaneous surgical treatment of morbid obesity and end-stage achalasia: a case report and review of literature

**DOI:** 10.3389/fsurg.2026.1767165

**Published:** 2026-03-24

**Authors:** Andrea Costantini, Luca Provenzano, Francesca Forattini, Matteo Pittacolo, Giulia Pozza, Luca Prevedello, Alice Albanese, Michele Valmasoni, Mirto Foletto, Renato Salvador

**Affiliations:** 1 Department of Surgery, Oncology and Gastroenterology, University of Padova, Unit of General Surgery 1, School of Medicine, Padova, Italy; 2 Department of Surgery, Oncology and Gastroenterology, University of Padova, Unit of Week Surgery, School of Medicine, Padova, Italy

**Keywords:** achalasia, case report, gastric bypass, obesity, pull-down technique, RYGB

## Abstract

**Background:**

End-stage achalasia is a challenging condition to deal with, due to severely impaired esophageal function. Laparoscopic Heller-Dor with pull-down technique improves the outcomes of these patients. Some patients with end-stage achalasia also suffer from severe obesity, thus requiring surgical treatment for both conditions. We report on a patient with severe obesity and an end-stage achalasia treated with pull-down laparoscopic myotomy in combination with Roux-en-Y gastric bypass (RYGB).

**Case presentation:**

A 42-year-old male with end-stage achalasia and severe obesity (kg 148, BMI 42.3 kg/m^2^) was proposed to the multidisciplinary group. Pre-operative assessment included symptom score, upper GI endoscopy, barium-swallow, high-resolution manometry (HRM), multidisciplinary bariatric evaluation. Two surgical teams with extensive experience in upper-GI and bariatric surgery were involved to plan the surgical treatment. The laparoscopic approach was carried out successfully without intraoperative complications. Firstly, approximately 10 cm of the lower mediastinal esophagus were isolated and pulled down into the abdomen. The esophagus was fixed to the crura with two non-absorbable stitches on each side, followed by an extensive Heller-myotomy. Then, a standard RYGB was carried out (100 cm alimentary- and BP-limb length). Gastrografin® x-ray was performed on POD 1, showing the verticalization of the organ's axis and the correct progression of the contrast through the anastomoses. The patient started a soft diet and was discharged on POD 4. At 6-months follow-up, the patient underwent HRM and 24h-pH monitoring showing a normal IRP and distal esophageal acid exposure. The symptom score was decreased to normal value and BMI was 31.5 kg/m^2^.

**Conclusion:**

To our knowledge, this is the first reported case of a combined approach to address end-stage achalasia and severe obesity with well-established and standardized operations. It proved to be feasible and effective to treat both clinical conditions. Further and formally designed studies are needed to confirm these results.

## Background

Achalasia and obesity are two clinical conditions that do not commonly co-occur (1). However, there is increasing epidemiological evidence regarding a relationship between these two conditions (2–4). First, achalasia is more prevalent in the surgery-naive obese population than in the general population (5–7). Moreover, obesity may occur following interventions for achalasia (8, 9). Furthermore, some authors suggest that bariatric surgery could be a risk factor for developing postoperative achalasia (10–13). Finally, the rising prevalence of obesity among the population and associated bariatric procedures suggest that the phenomena of achalasia and obesity will become increasingly prevalent (14). The aims of surgical treatment for achalasia patients are to improve quality of life and symptoms' relief (dysphagia, regurgitation and weight loss especially). Otherwise, the aims of surgical treatment in obese patients are to improve quality of life, promote healthy weight loss and reduce obesity-related co-morbidities. Currently, few data are available to guide the optimal timing of surgical approach to achalasia associated with severe obesity, mainly due to the rare occurrence of both conditions in clinical practice. However, a recent metanalysis highlighted the need to consider both diseases simultaneously when planning surgery for these patients (15).

End-stage achalasia is a rare condition in which the esophagus acquires a sigmoid shape and an augmented diameter (>6 cm) (16), representing about 5% of the achalasia patients undergoing laparoscopic Heller-Dor (LHD) (17). In these cases, laparoscopic myotomy is less effective than in early stages of the disease, and the need for esophagectomy is always around the corner. Recent studies, however, demonstrated that LHD with associated “pull-down” technique, is very successful in the treatment for these patients (18, 19). On the other hand, bariatric surgery (BS) is considered the most effective treatment to achieve durable weight loss and comorbidities resolution in subjects with severe obesity (20). Roux-en-Y-gastric bypass (RYGB) is considered the procedure of choice in this clinical setting, as the operation can address both obesity and EGJ disfunction (21).

At present, no data regarding the best treatment of end-stage achalasia associated with severe obesity are available. In this paper, we describe a case of a patient with morbid obesity and end-stage achalasia who had undergone a combined operation at our Center.

## Case presentation

A 42-year-old obese male described the beginning of his symptoms, based on dysphagia, regurgitation, retrosternal pyrosis and thoracic pain, 10 years ago, with an initial Eckardt score of 7. In his past clinical story, there were: hypertension on therapy with angiotensin-converting-enzyme inhibitors and an admission to the hospital due to a suspected transient ischemic attack (not confirmed). No surgical operations and allergies were reported; his medications were limited to a PPI and one ACE-inhibitor a day. He was 187 cm tall, and his weight was 148 kg, with a BMI of 42.3 kg/m^2^ (III grade obesity). He smoked 10–12 cigarettes per day for 17 years and his alcohol intake was about 1 alcohol unit (AU) on social occasions only.

In 2011 he underwent an endoscopy that showed motor anomalies of the esophagus, with chronic gastritis with the presence of H. pylori at pathological samplings. An esophageal manometry gave the final diagnosis of achalasia. Due to these findings, the patient underwent a successful pneumatic dilation with Rigiflex® 35 mm in 2011.

**Figure 1:**
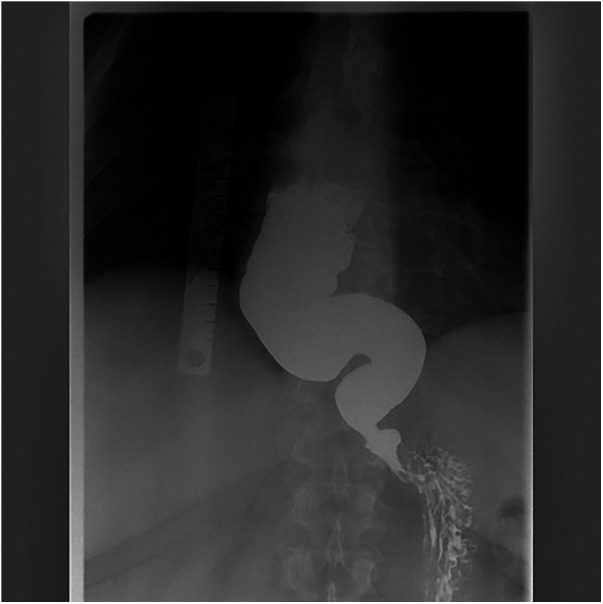
Preoperative barium swallow of the patient, demonstrating end-stage achalasia.

Following the 2011 dilation, symptoms remained largely controlled for several years; meanwhile, body weight remained high and tended to increase over time, with no clinically relevant weight loss. He was evaluated for BS at another hospital, with indication for a laparoscopic sleeve gastrectomy (LSG). However, during the preoperative evaluations, the patient complained about the recurrence of esophageal symptoms. So, he repeated a timed-barium swallow in 2022, where an abnormally dilated, sigmoid esophagus (end-stage diseases-radiological stage IV) was found (Figure 1). Notably, despite recurrence of dysphagia and regurgitation, the patient did not experience clinically relevant weight loss.

In preparation for the bariatric surgery, the patient underwent another cycle of pneumatic dilations at our Centre, with only a partial resolution of the symptoms (occasional dysphagia, thoracic pain and regurgitation, while body weight remained substantially stable, with an Eckardt score of 3). The patient underwent a pre-operative assessment with upper GI endoscopy, barium-swallow x-ray, and high-resolution manometry (HRM). Considering symptoms and the radiological stage of achalasia, the patient was evaluated for surgical intervention of LHD plus the pull-down technique. There were however doubts whether this operation would be feasible on a patient with a bariatric treatment program, if there was the possibility to treat both conditions at the same time and which surgical approach would be the best. The case was then discussed at a multidisciplinary meeting, involving two surgical teams with extensive experience in upper-GI and bariatric surgery, that concluded with the indication for a combined treatment of laparoscopic Heller myotomy (LHM) plus “pull-down” technique and a Roux-en-Y gastric bypass (RYGB).

**Figure 2:**
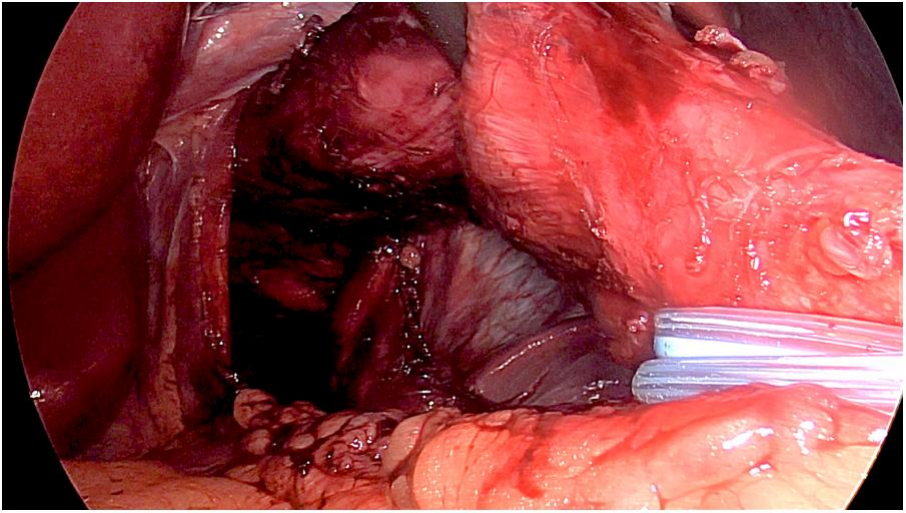
The pull-down technique. The esophagus is isolated from the pillars, and the dissection is extended about 10 cm in the mediastinum to take the esophagus in the abdomen, thus verticalizing the organ's axis.

**Figure 3:**
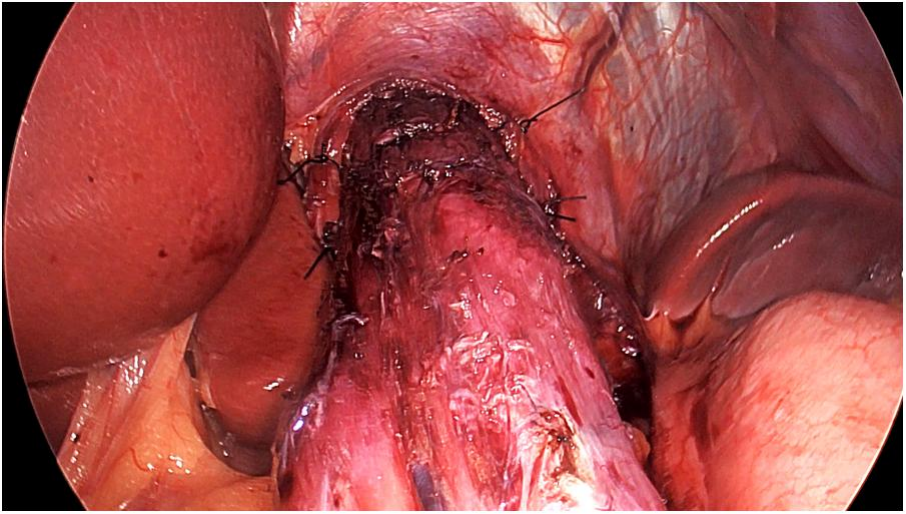
The surgeon applies 2 or more stitches on each side of the esophageal wall to anchor it to the pillars and to stabilize the straightening.

After that, in 2024 the patient underwent the combined operation, that consisted of:

- *Treatment of achalasia*. After freeing the lower esophageal attachments and circling the gastroesophageal junction using a string, approximately 10 cm of the lower mediastinal esophagus was isolated upward (Figure 2) pulling down the esophagus. The esophagus was then fixed with 2 stitches applied on each side of the esophageal wall and tied to the diaphragmatic pillars (Figure 3), thus verticalizing the organ's axis. After that, a Heller myotomy was performed following our standard technique (18), extending that for 10 cm in the esophageal side and 3 cm in the gastric side. Despite our standard technique for pull-down LHD, we avoided performing the Dor fundoplication to leave the gastric fundus intact for the creation of the gastric pouch for the RYGB. However, since myotomy without fundoplication may increase gastroesophageal reflux, RYGB was also expected to partially compensate this issue thanks to its known anti-reflux effect.
- *Treatment of obesity*. The jejunum was divided 100 centimeters distal to the ligament of Treitz using a stapler, leaving the proximal jejunum on the patient's right side. The Roux limb was passed anteriorly to the transverse colon. Then, with a stapler (Tri-Staple™, Medtronic, Minneapolis, USA), a gastric pouch with a capacity of approximately 30 mL was created. A latero-lateral anastomosis between the Roux limb and the proximal gastric pouch was then created, followed by the jejunojejunostomy.
- At the end, both the myotomy and the anastomoses were checked endoscopically.

No complications were detected during the postoperative course. On the 1st postoperative day (POD), the patient underwent a Gastrografin® swallow x-ray that showed the correct flow of the contrast through the cardia without any sign of leakage through the two anastomoses. The patient started to drink the same day; he then began oral feeding the following day and was dismissed on 4th POD.

**Figure 4:**
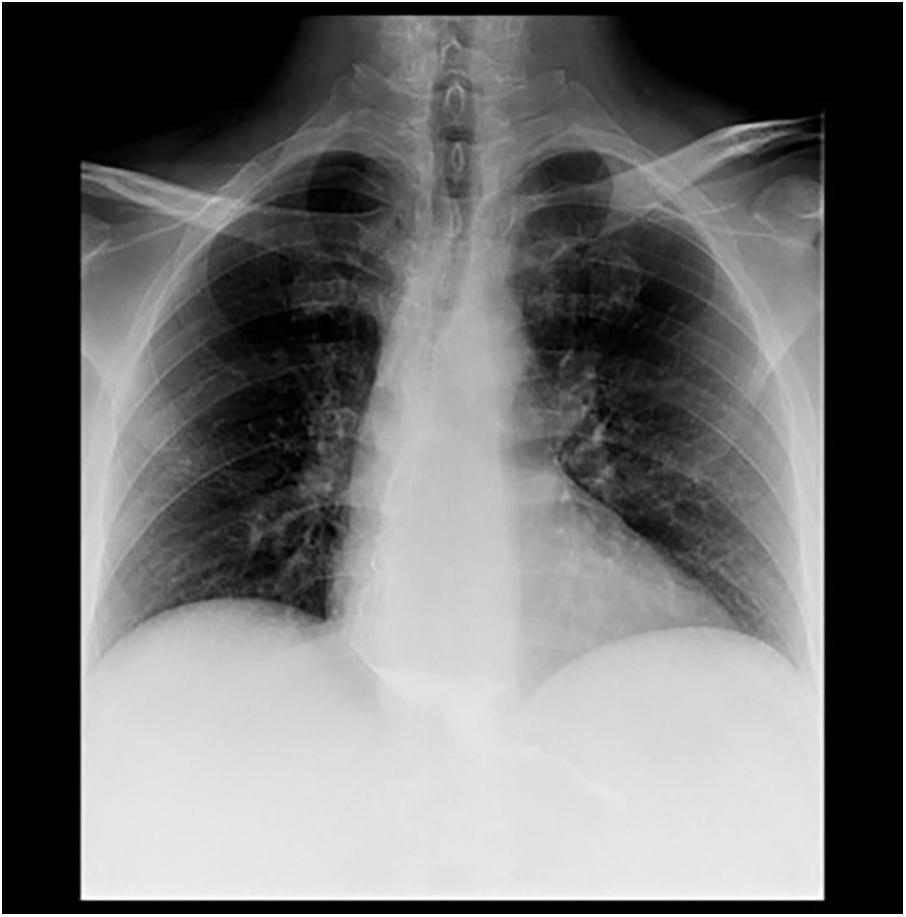
The postoperative barium swallow of the patient, one month after the surgical operation.

The follow-up was then regular. One month after the operation, the patient was satisfied with the operation, he only complained about occasional regurgitation after full-meal intake, and he started to lose weight (as the result of the bariatric operation). The barium swallow at one month showed an excellent flow of contrast with a good pull-down of the esophagus (Figure 4). At 6-months follow-up, the patient underwent HRM and 24h-pH monitoring showing a normal IRP and distal esophageal acid exposure. The symptom score decreased to normal value and BMI was 31.5 kg/m^2^.

**Figure 5:**
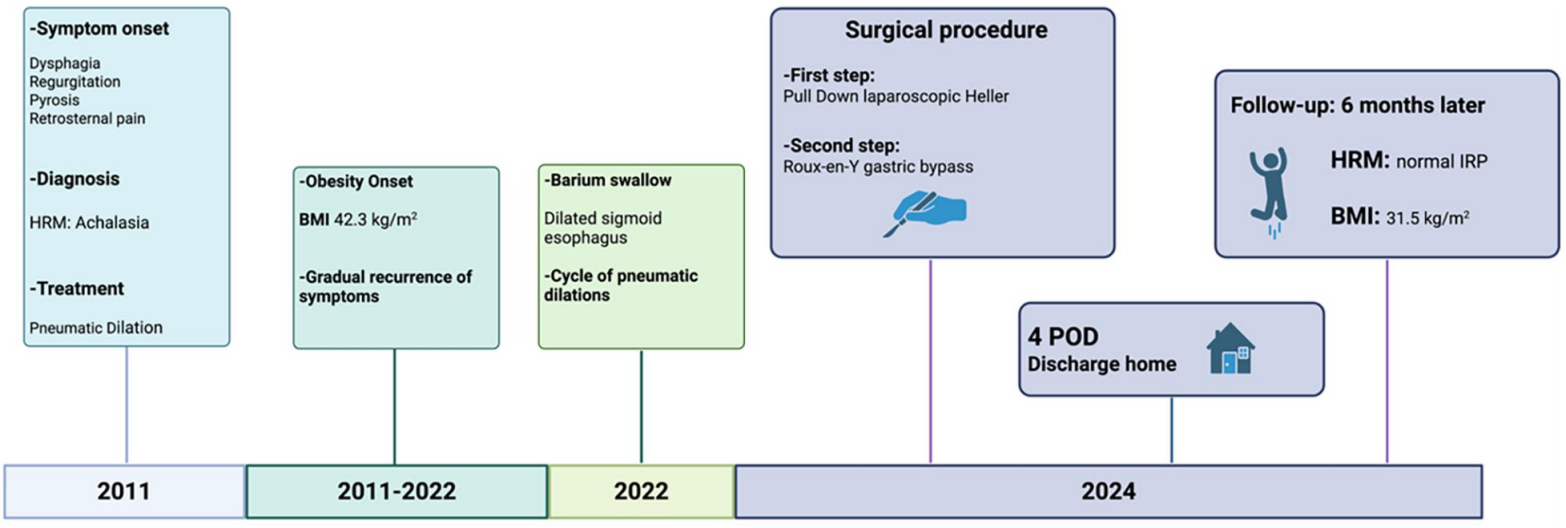
Timeline of the clinical case, reporting the key events from initial presentation to the most recent outpatient follow-up.

A timeline of the clinical case is presented in Figure 5.

## Discussion

Achalasia is a rare neurodegenerative disease affecting esophageal motility (22), with main symptoms consisting of dysphagia, regurgitation, chest pain and weight loss. In our case, the expected weight loss was not observed; on the contrary, the patient developed and maintained severe obesity despite long-standing achalasia. Therefore, the association of this rare disease with obesity seems like nonsense. However, with the epidemic of obesity throughout the world in the last decades (23), this association is sometimes reported (15). The reasons for this may be various. First, achalasia is more prevalent in the surgery-naive obese population than in the general population (5–7). Secondly, obesity may manifest following interventions for achalasia (8, 9). Another point is that bariatric surgery appears to be a risk factor for post-operative achalasia (10–13). Moreover, the rising incidence of obesity and associated bariatric procedures suggest that the phenomena of achalasia and obesity will become increasingly prevalent (14). Finally, a patient with achalasia may find extreme difficulty in swallowing healthy food like vegetables and fruits, preferring high-caloric semi liquid foods or junk foods.

All the available treatments for achalasia are palliative and aim at symptoms' relief, cutting or disrupting the muscular layers of the lower esophageal sphincter. To date, the treatments are either endoscopic with graded pneumatic dilations or peroral endoscopic myotomy (POEM), or surgical with LHM (24). These are highly effective in treating early stages of the disease (radiological stages I to III), that represent the majority of surgical cases (17). In particular, laparoscopic Heller-Dor provides satisfactory symptom improvement in 80%–85% of patients as long as and even longer than 10 years after surgery (17) and it is still considered the most effective long-term treatment. Albeit rarely, achalasia may progress to end-stage disease, as in our case. The treatment of end-stage achalasia is more cumbersome, with dilations (24), POEM (25) or even laparoscopic myotomy (17) resulting less effective than in earlier stages. Recently, the addition of the “pull-down” technique to the laparoscopic myotomy (18, 26) improved the outcome of surgery with comparable results between radiological stage IV achalasia patients and the ones with radiological stage I-III.

The choice of the best associated treatment for obesity and achalasia may be debatable. Commentaries from Wesp et al. and Shenfine drew the conclusion that simultaneous procedures for both pathologies were feasible (3, 4). Additionally, the management of achalasia following RYGB was explored by Aiolfi et al., which supported either LHM or POEM (11). Moreover, a recent metanalysis demonstrated that LHM and RYGB were the most common approaches for concurrent treatment of achalasia and obesity (15). Two aspects are crucial: choosing the most effective achalasia treatment for end-stage disease and selecting a bariatric procedure that does not worsen reflux. In stage IV achalasia, LHM with “pull-down” may be preferable to POEM, since POEM is less predictable in advanced disease and cannot be combined with fundoplication. Among bariatric options, LSG may exacerbate reflux, especially after myotomy, whereas RYGB is generally considered the best option to minimize postoperative reflux. Indeed, RYGB minimizes post-myotomy gastroesophageal reflux (27): this is achieved through the creation of a small gastric pouch, exclusion of most acid-producing gastric mucosa, decreased intra-gastric pressure, improved gastric drainage, and prevention of bile reflux (3). Moreover, both procedures utilize the same patient positioning and laparoscopic port placement and thus can be conveniently performed together (3). In the end, and most importantly, the need for an esophagectomy in end-stage achalasia must always be taken into consideration, both for the surgical failure of myotomy and the higher risk of end-stage achalasia patients to develop carcinomas of the esophagus (1). Evidence of an increased risk of developing esophageal cancer, both SCC and adenocarcinoma, has been described by studies examining large cohorts of patients (28–30). In our experience, 2 out of 94 patients (2.1%) with end-stage disease that underwent LHD with pull-down developed an SCC (19). Of note, 1 patient refused to undergo periodic endoscopies. Therefore, even if not fully accepted by the scientific community (24), we think that an endoscopy every 2 years after surgery can effectively exclude the possibility of long-term complications and allows early detection of any neoplastic degeneration (17). RYGB retains the native stomach to be used for future conduit formation, and several cases of esophagectomy using the stomach for reconstruction after bariatric surgery have been already reported (23).

Other combinations of bariatric and achalasia surgeries have significant limitations. For example, LHM and single anastomosis gastric bypass predispose to gastroesophageal bile reflux (31). LHM and LSG are synergistically refluxogenic (27, 32) and, above all, LSG precludes the stomach from future reconstruction after an eventual esophagectomy. LHM and gastric banding are inherently counter-productive, as banding will likely reverse the effect of the myotomy. POEM is a good alternative to LHM but, as reported, its efficacy in end-stage disease may be less rewarding than in early stage of the disease; moreover, it unnecessarily adds complexity to any bariatric procedure.

All these considerations must be kept in mind when approaching a patient with end-stage achalasia and morbid obesity. The treatment of both diseases requires particular care and should be planned in centers with extensive experience with both diseases. This requires the choice of the treatment for end-stage achalasia that provides the best long-term outcome of symptoms. At present, this is represented by LHM with associated “pull-down” of the redundant esophagus. The need for a future esophagectomy (for esophageal cancer or progression of the disease) must not be underestimated, however. Therefore, the choice of the complementary bariatric surgery should be reserved to procedures very effective in the weight reduction without causing other esophageal symptoms (i.e., reflux) and not compromising the possibility to use the stomach as a conduit after esophagectomy. Of the various bariatric surgeries, only RYGB fulfills these requirements.

## Conclusion

This described approach proved to be feasible, safe and effective to treat both clinical conditions. The case we presented illustrated the difficulties that surgeons face in treating end-stage achalasia and morbid obesity together. In our opinion, the key points are to offer the best conservative treatment that allows long-term results from the esophageal part and an effective surgical treatment for morbid obesity, that preserves most of the patient's organs, should they be needed for esophageal reconstruction.

## Patient's perspective

The patient expressed great satisfaction with the combined surgical approach, describing a clear improvement in swallowing and daily comfort. He appreciated the coordinated management of both conditions and reported a gradual return to his usual activities during follow-up.

## Data Availability

The original contributions presented in the study are included in the article/Supplementary Material, further inquiries can be directed to the corresponding author.

## Author's note

This work has been previously presented at the SAGES, 12–15, March 2025.
